# Association of Apolipoprotein E Gene Polymorphism with Lipid Profile and Ischemic Stroke Risk in Type 2 Diabetes Mellitus Patients

**DOI:** 10.1155/2021/5527736

**Published:** 2021-03-26

**Authors:** Ni Putu Tesi Maratni, Made Ratna Saraswati, Ni Nyoman Ayu Dewi, I Wayan Putu Sutirta Yasa, I Putu Eka Widyadharma, Ida Bagus Kusuma Putra, Ketut Suastika

**Affiliations:** ^1^Doctoral Program of Medical Sciences, Faculty of Medicine, Udayana University, Bali, Indonesia; ^2^Department of Internal Medicine, Faculty of Medicine, Udayana University, Sanglah General Hospital, Bali, Indonesia; ^3^Department of Biochemistry, Faculty of Medicine, Udayana University, Bali, Indonesia; ^4^Department of Clinical Pathology, Faculty of Medicine, Udayana University, Sanglah General Hospital, Bali, Indonesia; ^5^Department of Neurology, Faculty of Medicine, Udayana University, Sanglah General Hospital, Bali, Indonesia

## Abstract

**Background:**

Altered lipid profiles have consistently been linked to cerebrovascular events. Ischemic stroke (IS) was a common comorbid condition established in type 2 diabetes mellitus (T2DM). The apolipoprotein E (ApoE) gene which has a notably critical function in lipoprotein metabolism is believed as one of the potential candidate genes susceptible to IS complications in T2DM. This research aimed to determine the association of apolipoprotein E gene polymorphism with lipid profile and IS risk in T2DM patients.

**Methods:**

This case-control study involved a total of 60 diabetic participants divided into two groups with and without IS. ApoE was genotyped using PCR and sequencing analysis.

**Results:**

The most predominant genotype observed in 27 participants (45%) was E3/E3. Lower levels of high-density lipoprotein cholesterol (HDL-C) were found in *ε*2 carriers (*p*=0.003; 95% CI −23.35–−4.89) and *ε*4 carriers (*p*=0.019; 95% CI 1.38–14.55) compared to *ε*3 homozygotes. Total cholesterol (TC), triglyceride, and low-density lipoprotein cholesterol (LDL-C) levels had no association with ApoE gene polymorphism in this study. ApoE gene polymorphism was not related to IS in T2DM (*p*=0.06; adjusted OR: 4.71; 95% CI 0.93–23.79).

**Conclusions:**

ApoE *ε*2 and *ε*4 carriers were associated with lower levels of HDL-C. No association was identified between ApoE gene polymorphism and IS in T2DM patients.

## 1. Introduction

Type 2 diabetes mellitus (T2DM) is a complicated endocrine and metabolic disease, in which chronic hyperglycemia is provoked by peripheral insulin resistance and impaired release of insulin [1]. With the growing prevalence of overweight and obesity, concern has risen about an upsurge in the T2DM epidemic globally at an alarming rate [2]. The International Diabetes Federation (IDF) predicted that in 2017 about 425 million people worldwide have diabetes (8.8% of the adult population aged 20–79 years) and about 352.1 million people (7.3%) experience impaired glucose tolerance (IGT). By 2045, these figures are estimated to increase to around 629 million and 587 million, respectively. About 79% of diabetic patients live in countries with low to medium incomes. Indonesia is the sixth country with the greatest diabetes prevalence in the world, affecting around 10.3 million people in 2017 [3].

Long-term complications of T2DM, including microvascular and macrovascular diseases, are responsible as major sources of morbidity and mortality [4]. In addition to the increased diabetic nephropathy, neuropathy, and retinopathy, diabetes is also associated with stroke. T2DM patients have a fourfold greater chance of stroke compared to nondiabetic subjects [5]. Diabetic condition is also a major comorbidity factor that increases the recurrence and mortality rate from acute ischemic stroke (IS) [6]. Dyslipidemia may aggravate and promote atherosclerotic cerebral infarction in addition to the other risk factors like diabetes, hypertension, smoking habits, and family history of having cerebral infarction [7].

Apolipoprotein E (ApoE) gene and its proteins have an essential function in lipid metabolism and lipoprotein homeostasis [8]. ApoE is involved in the mechanism of facilitating the transport of triglycerides, phospholipids, cholesterol, and cholesterol esters across cells by intervening in the attachment and internalization processes of lipoprotein particles [9]. ApoE gene, mapped to chromosome 19, is noted to have a polymorphism comprising three main alleles: *ε*2, *ε*3, and *ε*4 and six distinct genotypes: E2/E2, E2/E3, E2/E4, E3/E3, E3/E4, and E4/E4 [10, 11].

ApoE gene polymorphism was established to be related to plasma lipid concentrations. The *ε*2 allele was linked with lower cholesterol levels and a lower possibility of having coronary heart disease, whereas the *ε*4 allele had a greater risk [12, 13]. The relation between polymorphism of the ApoE gene and stroke, however, remains uncertain. Many published articles reported about ApoE gene polymorphism involvement in stroke, but there were still conflicting results. ApoE *ε*4 allele is referred to as a predisposing factor for cerebral infarction [14–16] and increases the risk for different stroke subtypes (IS, intracerebral and subarachnoid hemorrhage) [17]. Contradictory results stated that no relationship was discovered between ApoE gene polymorphism and stroke [18–20]. In the current study, we intended to explore the association between ApoE gene polymorphism and plasma lipid parameters and the risk of having IS in T2DM as well.

## 2. Materials and Methods

### 2.1. Study Participants

The participants registered in this case-control study were recruited from the Diabetes Center Outpatients Clinic and Neurology Outpatients Clinic of Sanglah General Hospital. The study was conducted from February 1, 2020, to June 11, 2020. Before participation, a written informed consent was received from each patient. The Ethics Committee of the Faculty of Medicine in Udayana University approved the study protocol (138/UN14.2.2.VII.14/LP/2020). Enrolled subjects were grouped into two classes:

T2DM patients without IS included 30 patients diagnosed with T2DM as per the World Health Organization (WHO) criteria of fasting plasma glucose ≥126 mg/dL or 2-hour plasma glucose ≥200 mg/dL [21] or under oral diabetes medication and/or insulin.

T2DM patients complicated with IS included 30 subjects who fulfilled the WHO diagnostic criteria for diabetes or taking antidiabetic medication and complicated with IS. Diagnosis of IS was determined by symptoms and signs of focal neurological deficit arose from a vascular occlusive lesion with an abrupt onset and symptoms lasting longer than 24 hours and confirmed using noncontrast brain CT [22].

Both sexes and participants aged 30−78 years were included. Patients with any known renal disease, hepatic disease, malignancies, and autoimmune disease were excluded from this research. Physical and clinical data about age, sex, body mass index (BMI), blood pressure, diabetes duration, and smoking habits were recorded. Hypertension was determined by systolic blood pressure (SBP) ≥140 mmHg or diastolic blood pressure (DBP) ≥90 mmHg or taking antihypertensive drugs. Dyslipidemia was determined if either of the subsequent criteria is met: total cholesterol (TC) >200 mg/dL; triglycerides (TG) >150 mg/dL; low-density lipoprotein cholesterol (LDL-C) >130 mg/dL; and high-density lipoprotein cholesterol (HDL-C) <40 mg/dL [23].

### 2.2. Biochemical Analysis

After 12 hours of fasting, peripheral blood samples from studied participants were obtained in the morning. Fasting plasma glucose (FPG), hemoglobin A1C (HbA1C), TC, TG, LDL-C, and HDL-C were assayed on Cobas C501 autoanalyzer (Roche Diagnostics, Germany).

### 2.3. Apolipoprotein E Genotyping

Genomic DNA was isolated from peripheral blood using the GeneJET Genomic DNA Purification Kit (Thermo Fisher Scientific, USA) under the manufacturer's protocol. Amplification of the ApoE target sequences was performed by polymerase chain reaction (PCR) in the Veriti 96-Well Thermal Cycler (Applied Biosystems, USA). The forward and reverse primers used were 5′-TCCAAGGAGCTGCAGGCGGCGCA-3′ and 5′-GCCCCGGCCTGGTACACTGCCA-3′, respectively [24]. PCR was carried out: initial denaturation at 94°C for 5 min, followed by 40 cycles of denaturation at 94°C for 0.5 min, annealing at 69°C for 0.5 min, and extension at 70°C for 1.5 min, and also a final extension at 70°C for 10 min. Visualization of amplified PCR products was performed using gel electrophoresis in 0.8% agarose gel with FloroSafe DNA stain (First Base Laboratories, Malaysia). PCR products from these samples and single forward primer were sent to PT Genetika Science Indonesia (Banten, Indonesia) for DNA sequencing performed by ABI PRISM 3730*x*l DNA Sequencer (Applied Biosystems, USA).

### 2.4. Statistical Analysis

The collected data were evaluated using the Statistical Package for the Social Sciences version 17.0 application. The normality of data distribution was assessed by the Kolmogorov–Smirnov test. Numerical data were presented as mean value ± standard deviation (SD) for normally distributed data or median (interquartile range) for data followed a nonnormal distribution. Student's *t*-test or Mann–Whitney test was carried out to compare 2 groups. ANOVA test or Kruskal–Wallis test was used for more than 2 groups. Frequency and proportion were estimated for categorical variables and then tested by Chi-square test or Fisher's exact test. Multivariate logistic regression analysis was used to identify the association between disease and risk factors and expressed as adjusted odds ratio (OR). A statistically significant association is determined if *p*-value <0.05.

## 3. Results

### 3.1. Participants' Demographic Data

Participants' demographic and clinical data are provided in Table 1. The study involved 60 subjects classified into two categories which were T2DM complicated with IS (*n* = 30) and T2DM without complication (*n* = 30). Diabetic patients with IS had a higher level of SBP and DBP when compared to T2DM patients without complication (both *p*=0.001). A higher proportion of hypertension and smoking habits were found in T2DM with IS group compared to T2DM only patients (*p*=0.002 and *p*=0.008, respectively). T2DM + IS group had a lower level of 2-hour plasma glucose compared to the T2DM group (*p* < 0.001). Significantly higher levels of TC (*p*=0.027) and LDL-C (*p*=0.005) were observed among diabetic patients with IS than the T2DM group.

### 3.2. Genotype and Allele Frequencies of Apolipoprotein E

**Figure 1:**
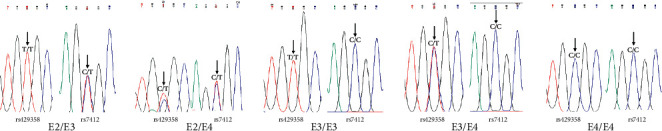
Apolipoprotein E genotyping on clinical DNA samples by sequencing.

The amplified DNA fragments had 218 bp that range across both ApoE polymorphic sites (rs429358 and rs7412). All ApoE genotypes results determined by sequencing analysis are depicted in Figure 1, with ApoE genotype and allele frequencies presented in Table 2.

In the total sample, E2/E3 genotype was 8 (13.3%), E2/E4 was 3 (5%), E3/E3 was 27 (45%), E3/E4 was 19 (31.7%), and E4/E4 was 3 (5%). Genotypes distribution based on the presence of IS complication among T2DM patients did not differ significantly between the two groups (*p*=0.382). ApoE allelic frequencies were found within the Hardy–Weinberg equilibrium in two groups. Overall, *ε*2, *ε*3, and *ε*4 alleles frequencies were 11 (9.2%), 81 (67.5%), and 28 (23.3%), respectively. No significant allele frequency difference was established between T2DM patients with and without IS (*p*=0.182).

### 3.3. Apolipoprotein E Gene Polymorphism and Lipid Parameters

ApoE genotypes were then classified aiming to see the relationship of ApoE gene polymorphism with lipid levels. ApoE genotypes were subdivided into three classifications, namely, *ε*2 carriers (E2/E2 and E2/E3), *ε*3 homozygotes (E3/E3), and *ε*4 carriers (E3/E4 and E4/E4) as shown in Table 3. Owing to the probable opposing influence of the *ε*2 and *ε*4 alleles on lipid concentrations, individuals having E2/E4 genotype (*n* = 3) were omitted from the following analysis.

Based on the results of the ANOVA test, a significant HDL-C level was found between the three groups (*p*=0.005). A lower HDL-C level was found in ApoE *ε*2 carriers in comparison with *ε*3 homozygotes: 33.25 vs. 47.37 mg/dL (*p*=0.003; 95% CI −23.35–−4.89). A lower significant level of HDL-C was also obtained in *ε*4 carriers compared to *ε*3 homozygotes: 39.41 vs. 47.37 mg/dL (*p*=0.019; 95% CI 1.38–14.55). The relation between the polymorphism of the ApoE gene and other determinant factors is also reported. Age, BMI, diabetes duration, sex, hypertension, or dyslipidemia were noticed to have no relationship with ApoE gene polymorphism (*p* > 0.05).

### 3.4. Apolipoprotein E Gene Polymorphism and Ischemic Stroke Risk in T2DM

Hypertension, smoking, high level of 2-hour plasma glucose (≥140 mg/dL), hypercholesterolemia (≥200 mg/dL), or high LDL-C level (≥130 mg/dL) were found to be substantially associated with IS in T2DM patients (*p* < 0.05) as shown in Table 4. After adjustment for other determinant conditions, multivariate logistic regression analysis was performed and indicated that hypertension was an independent risk factor for IS in T2DM (Table 5). Hypertension increased IS risk in T2DM patients by 5.01 folds (*p*=0.035; 95% CI 1.12–22.49). The *ε*4 allele was not an IS complication risk factor in T2DM (*p*=0.060; adjusted OR: 4.71; 95% CI 0.93–23.79).

## 4. Discussion

ApoE gene polymorphism in diabetic patients with or without IS in Balinese was first reported in our study. Our study reported that the most frequent genotype and allele were E3/E3 and *ε*3 homozygotes, respectively. We observed greater frequencies of the *ε*4 allele in total (23.3%) and smaller frequencies of the *ε*3 allele (67.5%) in the research participants in comparison with most other populations in previous articles. A study conducted in Thailand revealed greater frequencies of the non-*ε*3 allele like the *ε*4 allele [25]. This finding is indicated that the allelic distribution of the ApoE gene in Balinese is close to that in Thailand. Geographic distribution has a profound effect on the ApoE genotype and allele frequencies. Striking heterogeneity following a south-to-north gradient of ApoE allele frequencies was found in the European [26] and African populations [27]. The *ε*4 allele frequency heterogeneities were also found in Japan [28] and China [29].

This study showed that ApoE gene polymorphism was associated with plasma HDL-C concentrations. Both *ε*2 and *ε*4 carrier status appeared to be related to lower levels of HDL-C in comparison with the most common allele (*ε*3). These findings were supported by earlier studies that reported that the *ε*4 allele was associated with substantially lower plasma levels of HDL-C in the Thai population [30] and Spanish diabetic women [31]. Meta-analysis had shown that significantly smaller plasma levels of HDL-C were found in *ε*4 carriers in comparison with *ε*3 and *ε*2 carriers [13]. E3/E4 genotype was also found to have a relationship with lower levels of HDL-C in the Indian population [32]. Changes in the lipoprotein expression in T2DM are influenced by insulin resistance. The rate of HDL-C degradation increases during insulin resistance, but HDL-C production is not affected, which leads to a decrease in plasma HDL-C levels [33].

However, there were still inconsistent results from earlier studies regarding ApoE gene polymorphism's impact on other lipid parameters. Lower levels of HDL-C and also higher levels of TG were found in the CVD patients with T2DM who have *ε*4 allele [34]. E3/E4 genotype was related to higher concentrations of TC and non-HDL-C in all research participants and greater LDL-C levels in both T2DM and CVD groups [35]. In contrast, no relationship is found between ApoE gene polymorphism and other plasma lipid parameters (TC, TG, and LDL-C) in our study. The different impacts of ApoE gene polymorphism on the lipid concentrations can be affected by ethnic and environmental factors as indicated in these findings [36, 37].

The relationship of ApoE gene polymorphism with IS risk remains controversial. The *ε*4 allele was referred to be an important risk factor for cerebral infarction [14–16]. E3/E4 genotype and the *ε*4 allele were related to the incidence of IS among coronary heart disease patients receiving statin therapy [38]. On the contrary, we found that ApoE allele distribution has no association with IS risk in T2DM subjects. Similarly, several studies also suggested that although *ε*4 and *ε*2 carriers were associated with TC and TG levels, they were not associated with IS risk (19, 20) and patients' outcomes [39]. Our results also indicated that hypertension is the major independent risk factor for IS and has a significant impact than ApoE gene polymorphism on IS development. Blood pressure control was important for stroke progression in patients with diabetes [40].

## 5. Conclusions

This study indicates that both *ε*2 and *ε*4 carriers' statuses are associated with lower HDL-C levels. Unlike previous researches in other populations, we reported that there is no significant relationship between ApoE gene polymorphism and IS risk in T2DM. Additional researches are required to investigate whether genotyping of the ApoE gene is of value to screening complication progression in T2DM patients.

## Figures and Tables

**Table 1:** Demographic, clinical, and biochemical data of the participants.

Variable	T2DM + IS (*n* = 30)	T2DM (*n* = 30)	*p*
Age (years)	57.3 ± 9.63	57.47 ± 9.31	0.946
Sex (male/female)	17/13	18/12	0.793
BMI (kg/m^2^)	25.43 (22.96–27.47)	25.58 (23.73–28.12)	0.344
SBP (mmHg)	144 ± 24.86	125.67 ± 15.69	0.001^*∗*^
DBP (mmHg)	90 (80–100)	80 (70–80)	0.001^*∗*^
Hypertension	20 (66.7%)	8 (26.7%)	0.002^*∗*^
Diabetes duration (years)	4 [2–6]	3 [2–5]	0.688
Smoking	17 (56.7%)	7 (23.3%)	0.008^*∗*^
Dyslipidemia	25 (83.3%)	22 (73.3%)	0.347
FPG (mg/dL)	157.23 ± 53.59	160 ± 46.85	0.832
2-hour plasma glucose (mg/dL)	167.07 ± 52.24	224.33 ± 60.14	<0.001^*∗*^
HbA1C (mg/dL)	7.2 (6.5–9.2)	7.55 (6.8–8.8)	0.583
TC (mg/dL)	201.5 (172–252)	174.5 (144–202)	0.027^*∗*^
TG (mg/dL)	113 (101–167)	128.5 (95–194)	0.912
LDL-C (mg/dL)	143 (120–189)	116 (91–143)	0.005^*∗*^
HDL-C (mg/dL)	41.17 ± 12	42.77 ± 12.54	0.616

BMI: body mass index, SBP: systolic blood pressure, DBP: diastolic blood pressure, FPG: fasting plasma glucose, HbA1C: hemoglobin A1C, TC: total cholesterol, TG: triglycerides, LDL-C: low-density lipoprotein cholesterol, HDL-C: high-density lipoprotein cholesterol. ^*∗*^Statistically significant difference in comparison between two groups (*p* < 0.05).

**Table 2:** Apolipoprotein E genotype and allele frequencies of subjects.

Variable	T2DM + IS	T2DM	*p*
Genotype			0.382
E2/E2	—	—	
E2/E3	2 (6.7%)	6 (20%)	
E2/E4	2 (6.7%)	1 (3.3%)	
E3/E3	12 (40%)	15 (50%)	
E3/E4	12 (40%)	7 (23.3%)	
E4/E4	2 (6.7%)	1 (3.3%)	

Allele			0.182
*ε*2	4 (6.7%)	7 (11.7%)	
*ε*3	38 (63.3%)	43 (71.7%)	
*ε*4	18 (30%)	10 (16.7%)	

T2DM, type 2 diabetes mellitus; IS, ischemic stroke.

**Table 3:** Association of apolipoprotein E gene polymorphism with lipid levels and other metabolic-vascular risk factors.

Variables	*ε*2 carriers	*ε*3 homozygotes	*ε*4 carriers	*p*
Lipid profile				
TC (mg/dL)	175 (135.5–210.5)	202 (172.5–246.5)	175.5 (155–228)	0.423
TG (mg/dL)	158 (102–356.5)	115 (93–172.5)	130 (100–185)	0.439
LDL-C (mg/dL)	101.5 (79–146.5)	143 (119–171)	120 (109–161)	0.152
HDL-C (mg/dL)	33.25 ± 10.11^a^	47.37 ± 11.24	39.41 ± 12.08^b^	0.005^*∗*^

Metabolic-vascular risk factors				
Age (years)	58.13 ± 13.41	56 ± 8.87	58.05 ± 8.61	0.715
BMI (kg/m^2^)	26.38 ± 3.21	25.65 ± 2.96	26.39 ± 3.14	0.668
Diabetes duration (years)	6 ± 4.81	3.91 ± 3.56	3.5 ± 2.38	0.200
Sex (male/female)	5/3	13/14	14/8	0.548
Hypertension (%)	37.5	51.9	45.5	0.818
Dyslipidemia (%)	87.5	77.8	77.3	1.000

^*∗*^Statistically significant difference in comparison between three groups (*p* < 0.05). ^a^*p*=0.003 for *ε*2 carriers vs. *ε*3 homozygotes. ^b^*p*=0.019 for *ε*4 carriers vs. *ε*3 homozygotes.

**Table 4:** Bivariate analysis of variables related to ischemic stroke in T2DM.

Variables	T2DM + IS (*n* = 30) (%)	T2DM (*n* = 30) (%)	OR (95% CI)	*p*
Hypertension				
Yes	20 (71.4)	8 (28.6)	5.5 (1.81–16.68)	0.002^*∗*^
No	10 (31.3)	22 (68.8)		

Smoking habits				
Yes	17 (70.8)	7 (29.2)	4.3 (1.41–13.07)	0.008^*∗*^
No	13 (36.1)	23 (63.9)		

2-hour plasma glucose				
High	21 (42.9)	28 (57.1)	0.17 (0.03–0.85)	0.020^*∗*^
Normal	9 (81.8)	2 (18.2)		

TC				
High	16 (66.7)	8 (33.3)	3.14 (1.07–9.27)	0.035^*∗*^
Normal	14 (38.9)	22 (61.1)		

LDL-C				
High	19 (65.5)	10 (34.5)	3.46 (1.19–9.99)	0.020^*∗*^
Normal	11 (35.5)	20 (64.5)		

*ε*4 vs. *ε*3 carriers				
*ε*4 carriers	14 (63.6)	8 (36.4)	2.19 (0.69–6.93)	0.181
*ε*3 homozygotes	12 (44.4)	15 (55.6)		

^*∗*^Statistically significant difference (*p* < 0.05).

**Table 5:** Multivariate logistic regression of variables related to ischemic stroke in T2DM.

Variables	Adjusted OR	95% CI	*p*
Hypertension	5.01	1.12–22.49	0.035^*∗*^
Smoking habits	3.20	0.69–14.77	0.136
High 2-hour plasma glucose levels	0.55	0.07–4.35	0.574
Hypercholesterolemia	2.01	0.16–24.94	0.585
High LDL-C levels	4.38	0.35–54.66	0.251
*ε*4 carriers vs. *ε*3 homozygotes	4.71	0.93–23.79	0.060

^*∗*^Statistically significant (*p* < 0.05).

## Data Availability

The datasets used to support the findings in this study are openly available from the corresponding author upon reasonable request.
